# University Exams and Psychosocial Stress: Effects on Cortisol Rhythmicity in Students

**DOI:** 10.1111/cen.70083

**Published:** 2025-12-17

**Authors:** Filipy Borghi, Priscila Cristina da Silva, Elisângela Farias‐Silva, Fernando Canova, Aglecio Luiz Souza, Aline Barbedo Arouca, Dora Maria Grassi

**Affiliations:** ^1^ LABEEST‐Laboratory of Stress Study, Department of Structural and Functional Biology, Institute of Biology University of Campinas–UNICAMP Campinas São Paulo Brazil; ^2^ Unidade Metabólica, Faculdade de Ciências Médicas Universidade Estadual de Campinas (UNICAMP) Campinas São Paulo Brazil

**Keywords:** cortisol, hair, psychosocial stress, rhythmicity, saliva, undergraduate students

## Abstract

**Background:**

University exams are recognised as naturalistic stressors that may elicit psychosocial and physiological responses in students. This pilot study investigated the short‐ and long‐term effects of exam‐related stress on hypothalamic–pituitary–adrenal (HPA) axis activity, focusing on cortisol production and rhythmicity.

**Methods:**

Twenty‐seven undergraduate students (aged 18–24 years) from a biological sciences programme participated. Hair cortisol concentrations were analysed for October (non‐exam month) and November (exam month), while salivary cortisol was collected during 3 consecutive exam weekdays (Monday to Wednesday) at five daily time points to assess diurnal variation and the cortisol awakening response (CAR).

**Results:**

Hair cortisol levels were significantly higher in November than in October, suggesting greater cumulative HPA activation during the exam period. Salivary cortisol displayed a general diurnal pattern and CAR across the week. Morning cortisol values showed a nonsignificant numerical increase across days, though overall daily cortisol output (AUC) remained stable.

**Conclusion:**

Exam periods may be accompanied by elevated cumulative cortisol exposure while short‐term HPA rhythmicity remains preserved. The combined use of hair and salivary biomarkers offers a multidimensional and feasible approach to investigating acute and cumulative stress responses in students. Larger longitudinal studies incorporating behavioural measures are warranted to confirm these findings.

## 1 Introduction

Stress can be defined as a mismatch between external demands and the perceived ability to cope with the stressor [1, 2]. It is largely influenced by individuals' perceptions and cognitive appraisal, making it subjective in nature and difficult to predict objectively [2]. While stress responses are generally adaptive and play a vital role in enabling individuals to meet external challenges, chronic or overwhelming stress can have detrimental effects [3]. The neuroendocrine hypothalamus−pituitary−adrenal (HPA) axis is the primary mediator of the body's response to stress, with cortisol being its main downstream effector [4].

Systemic cortisol levels are highly variable and are affected by the circadian rhythm, acute stress situations and pulsatile secretion [5]. Traditional methods of measuring cortisol in saliva, blood or urine only provide snapshots of acute levels over relatively short sampling periods, typically 12−24 h, thus limiting the capacity to capture chronic stress responses [4, 5]. Furthermore, these assessments depend on strict adherence to sampling protocols by the patient, which can be challenging. In contrast, hair analysis offers a more stable and reliable biomarker for assessing chronic stress. It has been employed for decades to measure exposure to environmental toxins and drugs and has recently gained attention as a tool for evaluating long‐term HPA axis activity [5, 6, 7]. Hair cortisol analysis is particularly advantageous as it reflects endocrine activity over previous months, offering insights into past stress events [4].

Undergraduate students are frequently exposed to significant stressors throughout their university experience [8]. Research has shown that stressful situations in academic settings are associated with reduced academic performance, lower grade point averages, decreased graduation rates and higher rates of dropout, alongside heightened psychological distress [9, 10, 11]. The pressure to perform well during exam periods is a notable trigger of psychosocial stress, contributing to elevated cortisol release [12, 13]. Given the increasing levels of stress among undergraduates, this study aims to explore both the short‐ and long‐term effects of exam periods on the HPA axis in this population by measuring cortisol production over time.

Based on prior evidence linking academic stress with HPA axis activation, we hypothesised that the exam period would be associated with increased overall cortisol secretion, detectable in hair samples and that daily salivary cortisol patterns might differ from those observed under non‐exam conditions. This approach enables the simultaneous assessment of cumulative (hair) and diurnal (salivary) cortisol indices, providing a multidimensional view of stress‐related endocrine activity in students.

## 2 Methods

### 2.1 Subjects

Participants were undergraduate students aged 18−24 years, enroled in the Biological Sciences programme at a public university in Brazil. Recruitment occurred via classroom announcements and poster advertisements placed around campus. Volunteers received full information about the study aims and procedures and provided written informed consent before participating. The inclusion criteria required participants to be enroled full‐time or part‐time in the same academic curriculum, which helped minimise variability related to commuting or institutional stressors. Exclusion criteria included any current diagnosis of psychiatric or chronic physical illness, use of corticosteroids or psychoactive medications, smoking, excessive alcohol intake, shift work, irregular sleep schedules, pregnancy or recent major life events. All samples were collected during the final exam/test week at the end of the academic year. Final exams took place in the last week of November, and this period typically includes intense preparation, sleep reduction and academic pressure starting from early November. The study was conducted in accordance with the guidelines established in the Declaration of Helsinki, and ethical approval was granted by the Research Ethics Committee of the School of Medical Sciences/University of Campinas (CAAE: 41225614.8.0000.5404).

### 2.2 Hair Cortisol Samples

Twenty‐seven undergraduate students provided hair samples ranging from 3 to 5 cm in length. Strands were collected from the posterior vertex and cut as close to the scalp as possible, following Manenschijn et al. [14]. Hair cortisol was extracted and quantified using the protocol described by Meyer et al. [15]. Cortisol results were presented in nmol/L and derived from 40 mg of hair, resuspended in 0.2 mL of assay buffer and converted to pg/mg. Thus, the proximal 1 cm segment reflected cortisol accumulation during November (including the exam period), and the second cm reflected October, a month without exams.

### 2.3 Salivary Cortisol Samples

Twenty‐seven students collected saliva at home using Salivettes, stored under refrigeration. Samples were collected during 3 consecutive weekdays (Monday to Wednesday) of the final exam week in November. Samples were taken at 6 am (awakening), 6:30 am (30 min post), noon, 6 pm (pre‐dinner) and 11 pm (pre‐bedtime). Participants were instructed to follow normal routines while avoiding intense physical exercise, alcohol or other behaviours that might influence cortisol levels. Samples were centrifuged at 2800 rpm for 20 min at 4°C and analysed using a commercial ELISA kit (Diagnostics Biochem Canada Inc., Ref CAN‐C‐290) following Borghi et al. [8]. Cortisol values were expressed in nmol/L for each timepoint and also evaluated using the area under the curve (AUC).

### 2.4 Sample Size Justification and Sensitivity Analysis

Recruitment was constrained by the logistical burden of repeated saliva sampling and hair collection; therefore, this investigation used a convenience pilot sample. Post hoc sensitivity analysis using G*Power (v3.1) indicated that, with the achieved sample (*n* = 27, all participants provided both hair and salivary cortisol samples), the study had 80% power (*α* = 0.05) to detect approximately medium‐to‐large within‐subject effect sizes (Cohen's *d* ≈ 0.75–0.80). These calculations indicate the study is powered to detect medium/large effects but underpowered to detect small effects; therefore, null findings for small differences should be interpreted cautiously.

### 2.5 Statistical Analysis

For visual representation, data are presented as means ± standard error of the mean (SEM) to highlight the precision of the estimated means over time. To provide full information on variability, we report means ± standard deviation (SD), sample sizes and 95% confidence intervals for all measures in Supporting Information S1: Tables S1–S3. Normality was confirmed by the D'Agostino & Pearson's test. Paired or unpaired Student's *t*‐tests were performed for two‐group comparisons of parametric data, and Wilcoxon or Mann−Whitney tests were performed for nonparametric data. Within‐day timepoint comparisons were exploratory and interpreted with consideration of effect magnitude. One‐way ANOVA or Kruskal−Wallis test was used to compare cortisol measures across the 3 exam days, as appropriate based on normality assessment. All statistical analyses were conducted using GraphPad Prism version 10.00 for Windows (GraphPad Software, San Diego, California, USA). The level of significance was set at *p* < 0.05. Sensitivity analysis was performed using G*Power software to determine the smallest effect size detectable given the study's sample size.

## 3 Results

### 3.1 Hair Cortisol and Psychosocial Stress

**Figure 1:**
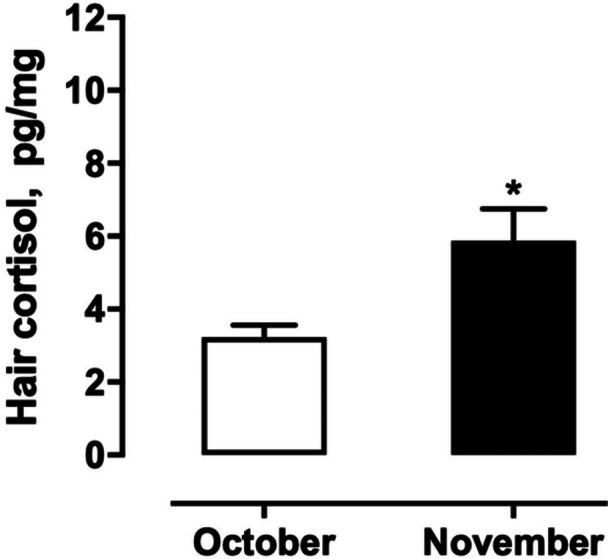
Hair cortisol levels in undergraduate students in October (a month without exams) and November (exam month, including preparation and assessment). Data are presented as mean ± SEM. *p < 0.05 within the same group.

Hair cortisol levels significantly increased from October (3.2 ± 0.4 pg/mg) to November (5.8 ± 0.6 pg/mg) (W = 174.0, *p* = 0.0067), suggesting a cumulative rise in chronic stress during the exam period (Figure 1). This elevation reflects sustained HPA axis activation likely due to prolonged academic pressure. These levels are consistent with previously reported values in healthy student and adult populations.

### 3.2 Salivary Cortisol

**Figure 2:**
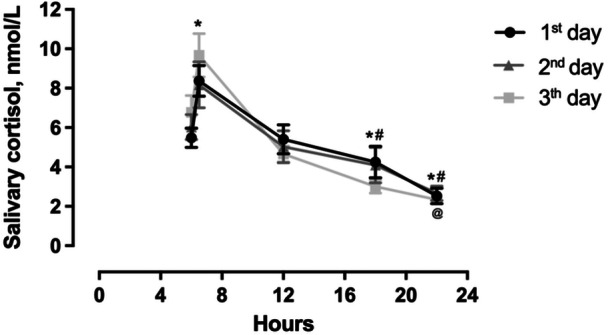
Cortisol rhythmicity from saliva samples collected at five different times of the day over 3 consecutive days: upon awakening (6 am), 30 min after awakening (6:30 am), before lunch (noon), before dinner (6 pm) and before bedtime (11 pm). Data are presented as mean ± SEM. *p < 0.05 versus 6 am within the same group; ^#^ *p* < 0.05 versus noon within the same group; ^@^ *p* < 0.05 for 6 pm versus 1st day 11 pm (1st Day only).

Salivary cortisol displayed a general diurnal pattern over the 3 consecutive weekdays, with peak levels at 30 min post‐awakening and progressive decline to evening nadir (Figure 2, Supporting Information S1: Table 2). Given the limited sampling frequency (four timepoints/day), we cannot definitively conclude that full circadian rhythmicity was preserved; nonetheless, the data are consistent with a typical diurnal decline. Sample sizes varied slightly across timepoints (*n* = 18–26) due to insufficient saliva volume in some samples or occasional missed collections, but all participants contributed data for at least 80% of scheduled timepoints. Within‐day comparisons confirmed preservation of the diurnal pattern with large effect sizes for the decline from morning to evening (*F* (10, 308) = 10.15, *p* < 0.001; Cohen's *d* = 0.77–2.07).

**Figure 3:**
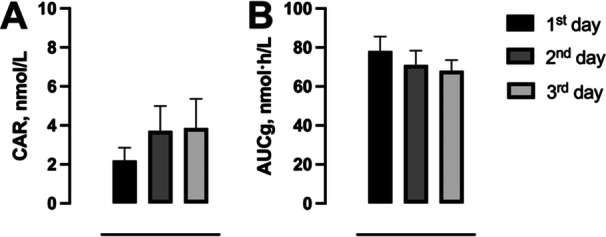
A) Cortisol awakening response (CAR) and B) Area Under the Curve (AUCg) in undergraduate students over different consecutive days. Data are presented as mean ± SEM. *p < 0.05 within the same group.

The cortisol awakening response (CAR) showed considerable inter‐individual variability and no statistically significant differences across the 3 exam days (Figure 3A, Supporting Information S1: Table 3A). Although mean CAR values were numerically higher on Day 3 than on Day 1, these differences were not statistically significant (*H* [2] = 0.69, *p* = 0.71).

Morning cortisol values (6 am and 6:30 am) showed a numerical increase across exam days, but this trend did not reach statistical significance after correcting for multiple comparisons (*F* (2, 55) = 0.86, *p* = 0.43). Evening concentrations (6 pm and 11 pm) remained stable or declined slightly (*F* (5, 127) = 2.03, *p* = 0.07). Consistent with these observations, the total daily cortisol output, quantified as the AUCg (nmol·h/L), did not differ between days (*F* (2, 74) = 0.58, *p* = 0.56) (Figure 3B, Supporting Information S1: Table 3B).

In summary, while hair cortisol indicated elevated cumulative stress, diurnal salivary cortisol patterns, CAR and AUC remained stable. A nonsignificant numerical increase in morning cortisol across days may reflect transient fluctuations, but no robust evidence of dysregulation was observed.

## 4 Discussion

This study examined both acute and cumulative cortisol measures to explore the effects of academic stress during an examination period. We observed changes consistent with increased HPA axis activity, although the magnitude of these effects should be interpreted cautiously, given the pilot nature of the sample.

Our findings partially supported the study hypotheses. Hair cortisol levels increased significantly during the exam month, suggesting cumulative HPA axis activation. However, salivary cortisol patterns should be interpreted cautiously given the limited sampling frequency (four timepoints/day). While a general diurnal decline was observed (morning peak to evening nadir), we cannot definitively conclude that full circadian rhythmicity was preserved due to the sparse sampling schedule. Similarly, total daily cortisol output (AUCg) remained stable across days, but this estimate is based on only four timepoints and may not fully capture intra‐day fluctuations.

Hair cortisol provides a valuable retrospective reflection of cortisol release over several months. In recent years, substantial evidence has accumulated regarding key aspects of hair cortisol measurements, including their validity as indicators of long‐term systemic cortisol concentrations, their reliability through repeated assessments and their robustness against various influences. Based on these findings, researchers have begun using cortisol measurements to address more specific questions about the role of stress in long‐term cortisol production and its impact on various health‐related conditions. Given its unique characteristics, hair analysis holds great promise for significantly enhancing our understanding of steroid hormones [4, 16, 17, 18].

The increased hair cortisol levels observed in November align with studies showing cumulative HPA activation in response to sustained academic demands [19, 20]. Exam‐related stress is not limited to the test session itself; rather, it builds progressively from the preparatory phase, often leading to prolonged sleep reduction, dietary changes and emotional strain. Hair cortisol reflects this cumulative exposure, supporting its role as a retrospective biomarker of chronic stress.

A major challenge in hair cortisol research is the wide inter‐individual variability observed even among participants of similar age within relatively small, non‐population‐based studies. This issue of ‘convenience sampling’ may limit the generalisability of our results, as it often leads to biases that may influence the outcomes. Nevertheless, our sample size and structure are comparable to other pilot studies in biomarker research [21, 22]. Future studies could address this limitation by utilising more representative sampling methods. Despite individual variability, the hair cortisol results in our study align with normative values in the literature. For example, studies in Mexican teachers and Icelandic women found average values of 6.0 and 4.7 pg/mg, respectively [23]. A Dutch study reported increasing levels from childhood to adolescence, reaching about 3.0 pg/mg by age 18 [24]. In Brazil, mothers and children had values of 5.6 and 7.8 pg/mg, respectively [25]. Our participants showed 3.2 pg/mg in October and 5.8 pg/mg in November, consistent with elevations expected during moderate psychosocial stress.

Biological techniques such as hair cortisol measurement are essential for understanding chronic stress and its mental health implications. Given the high prevalence of mental illness among university students [26], reliable biomarkers are increasingly necessary for early identification. In comparison to other groups, cortisol levels in this cohort are similar to those in older Canadian adults and lower than those in US medical students during clinical rotations [27, 28]. Despite the lack of international benchmarks for cortisol, this pilot study helps build foundational data for young adult populations, offering a reference point for future studies. Assay‐related variability may also have contributed to differences in absolute cortisol values across studies. Salivary cortisol concentrations can vary depending on the immunoassay or laboratory employed [29]. While the same method was consistently applied across all samples here, cross‐laboratory comparisons should be interpreted with caution.

The preserved salivary cortisol rhythms and stable CAR across the week suggest that participants maintained the capacity for normal dynamic HPA regulation during the exam period. Although CAR values showed considerable inter‐individual variability, no statistically significant systematic day‐to‐day changes were detected, consistent with the view that short‐term academic stress is insufficient to disrupt this component of cortisol rhythmicity. The CAR mechanism is independent of cortisol secretion throughout the day and is influenced by factors that are not yet fully understood. However, abnormalities in cortisol rhythms, whether in CAR or overall pattern, are associated with various diseases and psychosocial conditions [30]. A consistent CAR pattern, as observed in our study, may reflect psychological resilience or effective coping mechanisms. This interpretation is supported by previous research showing that individuals with intact CAR profiles tend to report better mental health outcomes under stress [31, 32]. However, psychological interpretations of these biological responses should be made with caution. While cortisol patterns may reflect stress adaptation, the absence of validated psychological measures (e.g., perceived stress, resilience, anxiety) limits our ability to triangulate physiological findings with self‐reported experience. Nonetheless, it is important to acknowledge that alterations in the CAR typically emerge over extended periods of repeated or chronic stress exposure. Several longitudinal investigations have shown that meaningful CAR changes are usually observed after weeks or months of persistent stress rather than across a few days of elevated demand [33, 34]. Therefore, our 3‐day assessment likely captured only transient or anticipatory responses rather than sustained dysregulation of the CAR mechanism specifically. The present findings should thus be viewed as preliminary observations within a limited timeframe.

The progressive increase in morning cortisol values across exam days, despite stable evening levels, may suggest mounting anticipatory stress as students prepared for consecutive exams [35]. However, this interpretation is speculative given the limited sampling window (3 days) and lack of pre‐exam baseline data. Additionally, our CAR assessment was constrained by the timing of the third saliva sample (noon), which occurred well after the typical CAR peak (~30–45 min post‐awakening). Thus, we cannot rule out subtle CAR changes that may have been missed. Future studies should include more frequent early‐morning sampling (e.g., 15, 30, 45 min post‐awakening) and longer observation periods to better capture anticipatory stress dynamics. We also did not record the exact timing of participants' exams, which may have introduced uncontrolled variability, as HPA activation could fluctuate depending on when during the day exams occurred. These limitations underscore our cautious interpretation that short‐term anticipatory effects in morning cortisol warrant further investigation in better‐controlled designs.

The divergence between elevated hair cortisol and relatively stable diurnal salivary indices suggests that cumulative exam‐related stress may manifest more clearly in long‐term biomarkers than in short‐term cortisol dynamics. This dissociation likely reflects differences in what each biomarker captures: hair cortisol integrates cortisol exposure over weeks, including both baseline and peak values across multiple stressful episodes throughout the exam preparation period, whereas daily salivary sampling captures only snapshots during the final exam week. By the time of saliva collection, students may have partially adapted to the exam routine, or the most intense stress may have occurred earlier in November during the preparation phase. Additionally, hair cortisol reflects cumulative exposure including nocturnal secretion and stress‐related pulses that may not be captured by five daily saliva timepoints [36]. Thus, elevated hair cortisol in the absence of marked daily dysregulation may indicate repeated transient HPA activation that accumulates over time without fundamentally disrupting circadian regulation. We did not examine correlations between hair and salivary cortisol, as these biomarkers capture distinct temporal windows (weeks vs. days) and biological constructs (cumulative exposure vs. diurnal regulation). Hair cortisol from November integrates approximately 4 weeks of HPA activity, whereas salivary samples represent only the final 3 days. A correlation analysis would conflate non‐overlapping time periods and would not be theoretically meaningful given the complementary, rather than redundant, nature of what each biomarker reflects.

This study involved a relatively small convenience sample, limiting statistical power to detect small effects. The absence of pre‐exam baseline sampling and validated psychological or behavioural assessments restricts our ability to link cortisol changes directly to perceived stress levels. Moreover, saliva sampling relied on self‐collection without electronic time verification, and we used an ELISA rather than a mass‐spectrometry approach, which may influence assay comparability. Although these constraints limit generalisability, the sample size is comparable to similar pilot studies using biomarker protocols with high participant burden [20, 37]. These constraints highlight the need for larger, longitudinal designs incorporating both physiological and psychometric measures to better characterise how academic stress affects HPA axis activity over time. Furthermore, as a pilot study with *n* = 27, our findings should be considered preliminary and require replication in larger cohorts.

This dual biomarker approach, combining hair and saliva, provides a nuanced view of both chronic and diurnal cortisol changes, capturing how stress unfolds temporally. This is especially relevant for at‐risk populations like university students, whose stress may fluctuate acutely and accumulate over time. While our sample size limits generalisability, our findings lay the groundwork for future investigations into stress profiles and early mental health interventions using biomarker‐guided screening.

The findings have potential implications for student well‐being programmes. Hair sampling could be used to identify students experiencing chronic stress accumulation, even when daily functioning appears preserved. The finding that students maintained normal diurnal rhythms and CAR despite elevated cumulative cortisol is reassuring, suggesting adaptive coping in this cohort. However, the progressive increase in morning cortisol across consecutive exam days may reflect subtle anticipatory fluctuations in morning cortisol during consecutive exam days. Educational institutions might consider reducing consecutive high‐stakes exams or implementing stress‐buffering interventions (e.g., mindfulness, sleep hygiene education), particularly during multi‐day exam periods, to prevent accumulation of anticipatory stress responses.

## 5 Conclusion

In summary, this pilot study demonstrates the feasibility of concurrently assessing hair and salivary cortisol to examine stress responses during an academic‐exam period. While preliminary, these findings may have implications for understanding how academic stress unfolds and could inform wellness strategies in higher education. However, given the pilot nature of the study, further research is essential to validate the effects observed here and explore their psychological and behavioural correlates. These preliminary results offer a foundation for future, larger‐scale investigations that combine biological and psychological metrics to better understand academic stress and student well‐being.

## Conflicts of Interest

The authors declare no conflicts of interest.

## Supporting information


**Supporting Note:** These tables provide complete descriptive statistics (mean, SD, SEM, 95% CI, and n) for all cortisol measures reported in the main text. SEM values are shown for visual consistency with the figures, while SD and 95% CIs reflect variability within the sample. **Supporting Table 1.** Hair cortisol concentrations (pg/mg) in undergraduate students during non‐exam (October) and exam (November) periods. Corresponds to Figure 1. Values are mean ± SEM (full descriptive statistics shown). **Supporting Table 2.** Diurnal salivary cortisol concentrations (nmol/L) across three consecutive days during the exam week. Corresponds to Figure 2. Samples collected at 06:00, 06:30, 12:00, 18:00, and 23:00 h. Values are mean ± SEM. **Supporting Table 3.** CAR (Cortisol Awakening Response; nmol/L) and Area Under the Curve (AUC; nmol⋅h/L) over three consecutive days during the exam week. Corresponds to Figure 3. Values are mean ± SEM.
