# Supplementary material for: University Exams and Psychosocial Stress: Effects on Cortisol Rhythmicity in Students
Source: Clin Endocrinol (Oxf). 2025 Dec 17;104(5):436–42. doi: 10.1111/cen.70083 (PMC13040516; doi:10.1111/cen.70083)
Supplement: Supplementary file 1 — Supporting Note: These tables provide complete descriptive statistics (mean, SD, SEM, 95% CI, and n) for all cortisol measures reported in the main text. SEM values are shown for visual consistency with the figures, while SD and 95% CIs reflect variability within the sample. Supporting Table 1. Hair cortisol concentrations (pg/mg) in undergraduate students during non‐exam (October) and exam (November) periods. Corresponds to Figure 1. Values are mean ± SEM (full descriptive statistics shown). Supporting Table 2. Diurnal salivary cortisol concentrations (nmol/L) across three consecutive days during the exam week. Corresponds to Figure 2. Samples collected at 06:00, 06:30, 12:00, 18:00, and 23:00 h. Values are mean ± SEM. Supporting Table 3. CAR (Cortisol Awakening Response; nmol/L) and Area Under the Curve (AUC; nmol⋅h/L) over three consecutive days during the exam week. Corresponds to Figure 3. Values are mean ± SEM. [file CEN-104-436-s001.docx]

**SUPPLEMENTARY MATERIAL**

**Supplementary Note:** These tables provide complete descriptive statistics (mean, SD, SEM, 95% CI, and n) for all cortisol measures reported in the main text. SEM values are shown for visual consistency with the figures, while SD and 95% CIs reflect variability within the sample.

**Supplementary Table 1.** Hair cortisol concentrations (pg/mg) in undergraduate students during non-exam (October) and exam (November) periods. Corresponds to Figure 1. Values are mean ± SEM (full descriptive statistics shown).

| **Figure 1** | October | November |
| --- | --- | --- |
| Number of values | 27 | 27 |
| Mean | 3.226 | 5.870 |
| Std. Deviation | 1.726 | 4.314 |
| Std. Error of Mean | 0.3322 | 0.8806 |
| Lower 95% CI of mean | 2.543 | 4.049 |
| Upper 95% CI of mean | 3.909 | 7.692 |

Note: Hair cortisol measured by ELISA; see Methods for assay details. Hair cortisol was significantly higher in November compared to October.

**Supplementary Table 2.** Diurnal salivary cortisol concentrations (nmol/L) across three consecutive days during the exam week. Corresponds to Figure 2. Samples collected at 06:00, 06:30, 12:00, 18:00, and 23:00 h. Values are mean ± SEM.

| **Figure 2** | 6 a.m. | 6:30 a.m. | noon | 6 p.m. | 11 p.m. |
| --- | --- | --- | --- | --- | --- |
| *1^st^ day* |  |  |  |  |  |
| Number of values | 18 | 21 | 21 | 26 | 20 |
| Mean | 5.485 | 8.371* | 5.404 | 4.253*^#^ | 2.532*^#@^ |
| Std. Deviation | 2.054 | 3.587 | 3.364 | 4.094 | 1.768 |
| Std. Error of Mean | 0.484 | 0.783 | 0.734 | 0.803 | 0.395 |
| Lower 95% CI of mean | 4.463 | 6.738 | 3.873 | 2.599 | 1.705 |
| Upper 95% CI of mean | 6.506 | 10.00 | 6.935 | 5.907 | 3.360 |
|  |  |  |  |  |  |
| *2^nd^ day* |  |  |  |  |  |
| Number of values | 20 | 21 | 24 | 21 | 21 |
| Mean | 6.023 | 8.993* | 5.027 | 4.094*^#^ | 2.656*^#^ |
| Std. Deviation | 2.854 | 5.406 | 3.994 | 4.138 | 1.727 |
| Std. Error of Mean | 0.638 | 1.180 | 0.815 | 0.903 | 0.376 |
| Lower 95% CI of mean | 4.687 | 6.535 | 3.341 | 2.210 | 1.870 |
| Upper 95% CI of mean | 7.358 | 11.45 | 6.713 | 5.978 | 3.442 |
|  |  |  |  |  |  |
| *3^rd^ day* |  |  |  |  |  |
| Number of values | 20 | 21 | 24 | 23 | 22 |
| Mean | 6.769 | 9.673* | 4.666* | 3.008*^#^ | 2.345*^#^ |
| Std. Deviation | 3.843 | 5.065 | 1.992 | 1.608 | 1.246 |
| Std. Error of Mean | 0.859 | 1.105 | 0.406 | 0.335 | 0.265 |
| Lower 95% CI of mean | 4.969 | 7.367 | 3.825 | 2.312 | 1.793 |
| Upper 95% CI of mean | 8.566 | 11.98 | 5.507 | 3.703 | 2.897 |

Note: Sample sizes vary (n = 18–26) due to insufficient saliva volume in some samples or occasional missed collections. All participants contributed data for at least 80% of scheduled timepoints. Salivary cortisol measured by ELISA; see Methods for details. Symbols indicate within-day exploratory comparisons demonstrating preserved circadian rhythm: *p < 0.05 vs. 6 a.m.; ^#^p < 0.05 vs. noon; ^@^p < 0.05 for 6 p.m. vs. 11 p.m. (1^st^ Day only). Effect sizes for circadian decline (Cohen's d) range from 0.77 to 2.07 (large to very large effects).

**Supplementary Table 3.** CAR (Cortisol Awakening Response; nmol/L) and Area Under the Curve (AUC; nmol⋅h/L) over three consecutive days during the exam week. Corresponds to Figure 3. Values are mean ± SEM.

| *A) Cortisol Awakening Response (CAR)* | *1^st^ day* | *2^nd^ day* | *3^rd^ day* |
| --- | --- | --- | --- |
| Number of values | 18 | 18 | 19 |
| Mean | 2.204 | 3.719 | 3.864 |
| Std. Deviation | 2.786 | 5.406 | 6.541 |
| Std. Error of Mean | 0.656 | 1.274 | 1.501 |
| Lower 95% CI of mean | 0.819 | 1.030 | 0.711 |
| Upper 95% CI of mean | 3.590 | 6.408 | 7.017 |

| B) Area Under the Curve (AUC) | *1^st^ day* | *2^nd^ day* | | *3^rd^ day* |
| --- | --- | --- | --- | --- |
| Number of values | 24 | 26 | 27 | |
| Mean | 78.19 | 71.01 | 68.08 | |
| Std. Deviation | 36.4 | 37.41 | 28.18 | |
| Std. Error of Mean | 7.441 | 7.336 | 5.422 | |
| Lower 95% CI of mean | 62.80 | 55.91 | 54.94 | |
| Upper 95% CI of mean | 93.58 | 86.12 | 79.23 | |
|  |  |  |  | |

Note: CAR calculated as the difference between cortisol at 06:30 h and 06:00 h (awakening); n = 18–19 depending on availability of paired samples. AUC calculated using the trapezoidal method from all five daily timepoints (06:00, 06:30, 12:00, 18:00, 23:00 h); n = 24–27 depending on completeness of daily sampling. No significant differences were observed across the three days for either CAR (Kruskal-Wallis test, H = 0.688, p = 0.709) or AUC (one-way ANOVA, F = 0.582, p = 0.561).
